# A bioeconomic performance index for comparison of an experimental intensive aquaponic system with tilapia and tomato versus aquaculture and hydroponics

**DOI:** 10.1038/s41598-026-49597-7

**Published:** 2026-04-24

**Authors:** Jesús Josafat De León-Ramírez, Leticia Félix-Cuencas, Samuel López-Tejeida, Juan Fernando García-Trejo, Carlos Francisco Sosa-Ferreyra

**Affiliations:** 1 Facultad de Ingeniería, Universidad Autónoma de Querétaro, El Marqués, Querétaro, Mexico; 2 Facultad de Medicina, Universidad Autónoma de Querétaro, Querétaro, Mexico

**Keywords:** Aquaponics, Bioeconomic assessment, Nutrient use efficiency, Circular economy, Sustainable food production, Biotechnology, Ecology, Ecology, Environmental sciences, Plant sciences

## Abstract

This study evaluates the bioeconomic performance index of an experimental intensive aquaponic system (IAS) integrating Nile tilapia (*Oreochromis niloticus*) and tomato (*Solanum lycopersicum*), in comparison with standalone aquaculture (AM) and hydroponic (HM) systems operated under identical greenhouse conditions. The systems were assessed at experimental scale over a 180-day production cycle using productive, environmental, and economic indicators integrated through a normalized Bioeconomic Performance Index (BPI) formulated as a benefit-to-resource ratio. Tilapia growth and feed conversion efficiency were comparable between IAS and AM, while water quality stability and nutrient use efficiencies were significantly higher in the integrated system (*P* < 0.05). Tomato yield was highest in the hydroponic system; however, crop production in the aquaponic system was achieved without synthetic fertilizers, relying exclusively on recycled aquaculture-derived nutrients. Although the IAS exhibited higher operating costs, mainly due to energy consumption associated with continuous water circulation and filtration, the combined production of fish and crops resulted in the highest net profit among the evaluated systems. The BPI indicated that the intensive aquaponic system achieved superior overall bioeconomic performance by balancing higher resource demand with gains in combined output and resource-use efficiency. Sensitivity analysis identified energy consumption as the dominant factor influencing bioeconomic performance, without altering system ranking. These results suggest that intensive aquaponics can represent a competitive alternative to conventional aquaculture and hydroponics when evaluated through an integrated bioeconomic framework, particularly in contexts prioritizing water conservation and nutrient recycling, while acknowledging trade-offs related to energy use and system complexity.

## Introduction

Aquaponics has gained increasing attention as an integrated food production strategy that combines recirculating aquaculture and hydroponics, enabling the reuse of nutrients and water within a single production system. By coupling fish and plant production, aquaponics aligns with sustainability and circular economy principles by reducing nutrient discharge, freshwater consumption, and dependency on synthetic fertilizers while generating multiple marketable products^1–3^. From a circular economy perspective, nutrients originating from aquaculture effluents are internally recycled and valorized as inputs for plant growth, closing material loops and reducing external resource inputs. These characteristics position aquaponics as a promising alternative to conventional single-component production systems, particularly in regions facing water scarcity and growing pressure on natural resources.

Some studies have compared aquaponic systems with standalone aquaculture or hydroponic systems, focusing on fish growth performance, plant yield, water use efficiency, and nutrient dynamics^4–6^. While these studies provide valuable insights, most assessments rely on isolated indicators analyzed independently. As a result, system-level trade-offs between productivity, resource-use efficiency, and economic performance are not always fully captured, especially under intensive production conditions characterized by high cultivation densities, where energy demand and operational costs may increase^3,7^.

Economic analyses further highlight this complexity. Although aquaponic systems can reduce fertilizer and water inputs, higher operational costs related to energy consumption and system management are frequently reported, leading to context-dependent economic outcomes^2,8^. This variability underscores the need for integrative assessment approaches capable of simultaneously accounting for productive, environmental, and economic dimensions within a unified analytical framework.

To address this gap, bioeconomic assessment tools have been proposed to integrate multiple performance dimensions into composite indicators that support decision-making in aquaculture and integrated agri-food systems^8,9^. However, comparative bioeconomic evaluations of intensive aquaponic systems relative to conventional aquaculture and hydroponics operated under comparable conditions remain limited, particularly those explicitly quantifying trade-offs between higher operational costs and gains in resource-use efficiency^7^.

Although an increasing number of studies have compared aquaponic systems with standalone aquaculture or hydroponic production, important knowledge gaps remain. Many existing comparisons are constrained by differences in system scale, management intensity, and boundary conditions, limiting the direct evaluation of trade-offs among productivity, resource efficiency, and economic performance. Moreover, most analyses focus on isolated productive or environmental indicators, with limited integration of techno-economic dimensions. As a result, the combined effects of nutrient recovery, resource efficiency, and operational costs particularly energy demand remain insufficiently quantified under intensive production scenarios, leaving the relative advantages and limitations of aquaponics incompletely resolved.

In this context, the present study proposes and applies a Bioeconomic Performance Index (BPI) as an exploratory, integrative system-level indicator that combines productive output, environmental efficiency, and economic performance within a single comparative framework. The aim of this study was to compare the bioeconomic performance of an experimental intensive aquaponic system integrating tilapia (*Oreochromis niloticus*) and tomato (*Solanum lycopersicum*) with conventional aquaculture and hydroponic systems operated independently under comparable conditions. We hypothesized that system integration would enhance water and nutrient use efficiency, and that the higher operational costs associated with aquaponics would be partially offset by the combined production of fish and crops, resulting in competitive overall bioeconomic performance despite increased energy demand. By doing so, this study addresses the lack of experimentally based, integrated bioeconomic comparisons of intensive aquaponic systems relative to standalone production models under equivalent operational conditions.

## Materials and methods

The study was carried out in a greenhouse at the Aquaculture Unit of the Amazcala Campus of the Autonomous University of Querétaro, Mexico (20º38 × 43.3´´ N, 100º25 × 10.3´´ W; 1,980 m.a.s.l.), from March to August 2022 to 2024. Three production systems were evaluated in a randomized block experimental design with three replications. Each block included: (1) an intensive tilapia-tomato aquaponic system IAS, (2) a conventional aquaculture module AM, and (3) a hydroponics module HM with conventional nutrient solution. All animal procedures were conducted in accordance with the relevant institutional guidelines and regulations for the care and use of laboratory animals. The experimental protocol was reviewed and approved by the Ethics Committee of the Faculty of Engineering of the Autonomous University of Querétaro (ID 10846). All methods are reported in accordance with the ARRIVE guidelines for animal research.

### System description

The experimental setup consisted of three production systems operated simultaneously under identical greenhouse conditions: an intensive aquaponic system (IAS), a conventional aquaculture module (AM), and a hydroponic module (HM). All systems were operated at an experimental scale and installed within the same greenhouse under passive ventilation, natural light, and without supplemental heating or CO₂ enrichment throughout a 180-day production cycle. The greenhouse area allocated to each system was 20.4 m² for the IAS, 7.2 m² for the AM, and 14.4 m² for the HM.

**Fig. 1:**
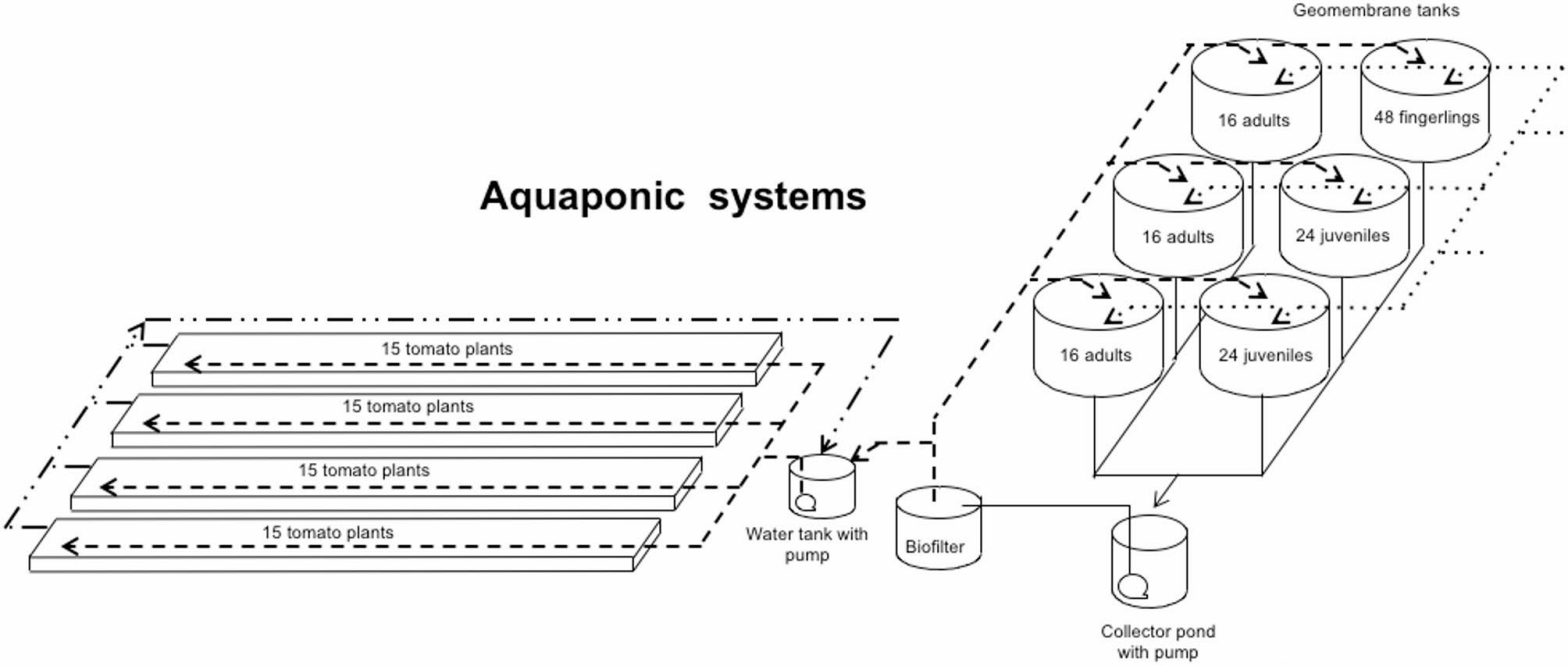
Operational layout of the experimental intensive aquaponic system (IAS) integrating fish and plant production.

The IAS functioned as a closed-loop system integrating fish and plant production (Fig. 1). It comprised six fish tanks (100 L each) connected to a common collection sump and filtration unit. Water was continuously recirculated using a submersible pump (12,000 L/h, 750 W) through a cartridge biofilter (BOYU^®^ EFU-13500, with integrated UV sterilizer) and returned to the fish tanks. A fixed volume of 100 L/day was diverted from the biofilter to the plant irrigation reservoir (equipped with a 45 W submersible pump) via a solenoid valve controlled by a timer; an equivalent volume was simultaneously replenished in the fish tanks to maintain constant system water balance. The plant component consisted of four growing beds (5 × 0.25 m each) filled with coconut fiber substrate and irrigated by drip emitters, with drainage water returned to the aquaculture circuit to ensure complete recirculation of water and nutrients.

**Fig. 2:**
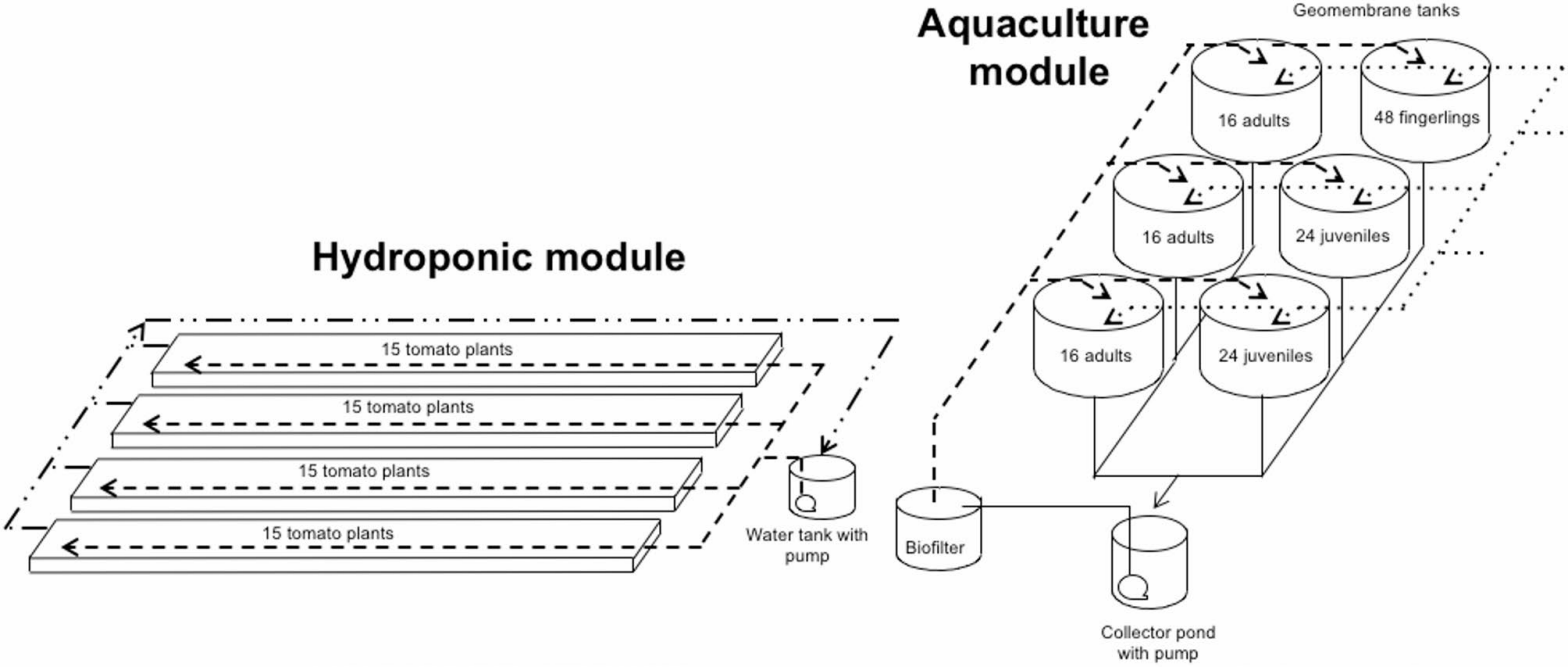
Operational layout of the experimental control modules: aquaculture module (AM) and hydroponic module (HM).

The AM served as the fish-only control system (Fig. 2) and was constructed using the same basic aquaculture components as the IAS, including tank number and volume, stocking density, filtration unit, and water recirculation scheme. It consisted of six independent fish tanks (100 L each) operated under identical hydraulic and management conditions to those of the aquaponic system, with the sole difference being the absence of any plant production unit. Similarly, the HM served as the plant-only control system (Fig. 2) and employed the same horticultural components used in the IAS, including growing bed dimensions, substrate type, and drip irrigation configuration. The hydroponic system was supplied by an independent nutrient solution reservoir and operated without any connection to the aquaculture component, thereby isolating plant production from fish-derived nutrient inputs.

### Experimental design

To evaluate the bioeconomic performance of an intensive aquaponic system (IAS) for tilapia (*Oreochromis niloticus*) and tomato (*Solanum lycopersicum*), a 180-day comparative experiment was conducted using a randomized complete block design. The study was carried out over three consecutive production cycles conducted in different years using the same experimental infrastructure and system configuration. These production cycles were treated as temporal repetitions of the same experimental design rather than as independent biological replicates. Consequently, year was not considered an independent experimental factor but a source of temporal variability, thereby avoiding pseudoreplication. Each system (IAS, aquaculture module: AM, and hydroponic module: HM) functioned as an independent experimental unit. In the IAS, tilapia was stocked at a target harvest density of 40 kg/m³, and tomato plants were established at a density of 8 plants/m². The hydroponic module (HM) supplied with a commercial nutrient solution formulated for hydroponic cultivation, applied at standard concentrations (mg/L): 150 N, 48 P, 216 K, 31 Mg, 125 SO₄, 3 Fe, 0.5 Mn, 1.5 Zn, 0.15 Cu, 0.5 B, and 0.1 Mo. The aquaculture module (AM) was operated as a recirculating system without plant integration and used the same fish density, water volume, tank configuration, and filtration capacity as the IAS. All systems were operated under identical greenhouse environmental conditions to ensure comparability among treatments. Natural daylight provided a seasonal photoperiod, and no artificial lighting was used. Ambient temperature and relative humidity were regulated passively through ventilation and shading and remained within ranges suitable for tilapia and tomato cultivation. Luminosity, humidity, and photoperiod were not manipulated as experimental variables and were common to all systems, minimizing potential confounding effects.

###  Biological material

For tilapia (*Oreochromis niloticus*) culture, both the IAS and the aquaculture module (AM) stocked fish at different physiological stages to achieve staggered culture. Each system included one tank with 48 fingerlings (approximately 5 g), two tanks with 24 juveniles each (approximately 50 g), and three tanks with 16 adult fish each (approximately 150 g), totaling 144 fish per unit (Table 1). Fish in both the IAS and AM were fed three times a day with commercial MaltaCleyton^®^ brand feed (Table 2), with rationing adjusted according to growth stage and biomass. The tilapia (*Oreochromis niloticus*) specimens used in the experiment were obtained from the Aquaculture Unit of the Autonomous University of Querétaro, Amazcala Campus.

Forty-day-old tomato plants (*Solanum lycopersicum*, var. Río Grande) were grown at a density of 8 plants/m²^10^. A total of 60 plants per unit (15 plants per bed) were planted in both the intensive aquaponic system (IAS) and the hydroponic module (HM). Plants in both the (IAS) and hydroponic systems (HM) were irrigated three times a day using drip irrigation, with volumes adapted to the phenological phase (vegetative, flowering, and fruiting) to maintain optimal substrate moisture (Table 3). In the HM, the nutrient solution was prepared weekly with the commercial Peters^®^ 5–11-26 fertilizer, maintaining the standard concentrations of macronutrients and micronutrients recommended for hydroponic tomato cultivation. In contrast, in the IAS, no external fertilizers were applied; all plant-available nutrients came from aquaculture effluent. No pesticides were applied in either system, as pest control was performed manually. Throughout the 180-day experiment, water quality parameters such as temperature, pH, dissolved oxygen, ammonium, nitrite, and nitrate were regularly monitored to ensure optimal conditions for both aquatic and horticultural components. The relatively high irrigation volume applied in the aquaponic system reflects internal water recirculation rather than net water consumption. Increased flow rates were intentionally used to ensure homogeneous nutrient distribution, prevent localized nutrient depletion in the root zone, and maintain adequate oxygen availability for plant roots.

**Table 1:** Distribution and biometric characteristics of tilapia (*Oreochromis niloticus*) across developmental stages: fingerlings, juveniles, and adults.

Productive stage	Initial weight (individual)	Number of fish	Number of tanks	Expected final weight (individual)	Expected final biomass
Fingerling	5.65 ± 0.12 g	48	1	50 g	2.4 kg
Juvenile	51.13 ± 2.73 g	24	2	150 g	3.6 kg
Adult	150,98 ± 8.21 g	16	3	250 g	4 kg

**Table 2:** Composition and schedule of the feeding plan used for tilapia (*Oreochromis niloticus*) based on MaltaCleyton^®^ commercial diets.

Stage	Weight range per fish	Protein	Lipid	Daily percentage of feed	Feeding times
Fingerling	5–20 g	45%	16%	8%	8:00 am (30%) 13:00 pm (40%) 18:00 pm (30%)
Fingerling	20–50 g	45%	16%	5%	
Juvenile	50–150 g	35%	3%	4%	
Adult	150–300 g	30%	3%	2%	

**Table 3:** Irrigation management schedule implemented during the experimental period, based on Mercado-Luna^11^.

Stage	Irrigation volume per plant	Schedules and irrigation ration
Vegetative	1.5 L	10:00 am (30%) 14:00 pm (40%) 16:00 pm (30%)
Flowering	2.4 L	
Fructification	3.6 L	
Maturation	2.4 L	

### Management of organisms

Fish growth was monitored biweekly through biometric sampling (weight and length). Based on the collected data, feeding rates were adjusted according to biomass. During biometric, fish were temporarily removed, measured, and returned to their respective tanks, minimizing handling time and stress. On days 60 and 120 of the production cycle, population restructuring was implemented to maintain a staggered growth strategy: adult fish were harvested; juveniles were redistributed among three tanks (16 individuals per tank); fingerlings were assigned to two tanks (24 individuals per tank); and 48 new fingerlings (initial average weight of approximately 5 g) were introduced into one tank. Fish yield (kg/m³) was calculated based on biomass harvested at each harvest event (days 60, 120, and 180). Yield values reported as mean ± standard deviation correspond to pooled data from three harvests per cycle across three consecutive production cycles (*n* = 9). For the sampling procedures, the fish selected for analysis were fasted for 24 h^12^ and subsequently euthanized under anesthesia as part of the final sampling procedures. The fish were anesthetized with a eugenol solution (100 mg L⁻¹) for approximately 3 min^13^. Harvested fish that were not subjected to analysis were transferred to the institutional aquaculture processing unit for standard commercial processing. All handling procedures were performed in accordance with institutional ethical guidelines to minimize stress and ensure animal welfare.

Tomato plant management followed a phenology-based protocol. During the vegetative stage, all plants were provided with vertical support (staking). During the flowering stage, daily manual pollination was performed following a standardized procedure applied uniformly across all experimental units, and regular pruning of axillary shoots and lower leaves was initiated. During fruiting, fruit thinning was performed, limiting bunches to a maximum of six fruits each to promote uniform development. Pruning was continued to maintain plant structure and vigor. At the ripening stage, tomatoes were harvested at maturity level 4, defined as 30–60% of the fruit surface showing pink or red color, according to the Camello classification criteria^14^. Tomato yield (kg/m^2^) was calculated from individual production lines. Four hydroponic or aquaponic growing lines per system were monitored in each production cycle. Yield values reported as mean ± standard deviation correspond to the pooled data from four lines across three consecutive production cycles (*n* = 12).

### Water quality

Water quality was regularly monitored to ensure adequate conditions for both the aquatic and horticultural components of the system. In the fish tanks, dissolved oxygen (DO), pH, and temperature were measured daily using a Hach^®^ HQ40d multiparameter meter. Nitrogen compounds were also analyzed weekly using a Hach^®^ DR6000 spectrophotometer, following the manufacturer’s standardized methods: nitrate (Method 8039), nitrite (Method 8507), and total ammonia nitrogen (TAN, Method 8038). In the plant irrigation system (IAS and HM), pH and DO were also recorded daily using the same multiparameter meter. Electrical conductivity (EC) was measured using a HANNA^®^ HI 98,130 conductivity/pH meter. Whenever necessary, the pH was adjusted to 6.0 with citric acid. All measurements were performed in triplicate, and mean values were used for subsequent analyses. The multiparameter probe (HANNA^®^ HI) were calibrated and periodically verified following the manufacturers’ procedures. Spectrophotometric analyses (Hach^®^ DR6000) were conducted using method-specific blanks and the internal quality control protocols embedded in the standardized Hach analytical methods.

### Productive performance

To evaluate and compare the performance of the intensive aquaponic system with conventional aquaculture and hydroponics, a comprehensive bioeconomic evaluation was conducted. This approach integrated a set of indicators reflecting the technical, environmental, and financial dimensions of each system, allowing for a multidimensional comparison of performance. The evaluated variables were grouped into three main categories: productive variables, representing biological efficiency and system performance; environmental variables, associated with resource use and ecological impact; and economic variables, related to cost structure and profitability.

#### Productive variables

Productive performance was evaluated for each organism (tilapia and tomato) in each system. The selected variables allowed for quantifying biomass accumulation, physiological growth efficiency, and survival at the end of the cycle. For tilapia, the following variables were evaluated: the total tilapia yield (Eq. 1), total weight gain (Eq. 2), daily weight gain (Eq. 3), feed conversion ratio (Eq. 4), protein efficiency (Eq. 5)^15^, specific growth rate (Eq. 6) and fish survival rate (Eq. 7)^16^. For tomato plants, the following variables were considered: total fruit yield (Eq. 8), dry weight (at flowering, fruiting and harvest stages) (Eq. 9), relative growth rate (Eq. 10) and survival rate (Eq. 11)^17^ and crop growth rate (Eq. 12)^18^. All measurements were recorded at regular intervals and standardized by plant or fish number and system volume or area, as appropriate.

1 $$\:\mathrm{Total\:Tilapia\:}\mathrm{Yield} \;\;\mathrm{TTY}\mathrm{\:(kg}{\text{}\mathrm{m}}^{\mathrm{-}\mathrm{3}}\mathrm{)}\mathrm{=\:}\frac{kilograms\:of\:tilapia}{Volume\:of\:water\:used\:{m}^{3}}$$

2 $$\:\mathrm{Total\:}\mathrm{Weight}\text{}\mathrm{Gain} \;\; \mathrm{TWG\:(g)=\:}\mathrm{Wf}\mathrm{\:-\:Wi}$$

Where: Wf is the final weight and Wi is the initial weight

3 $$\:\mathrm{Daily}\text{}\mathrm{Weight}\text{}\mathrm{Gain} \;\; \mathrm{DWG\:(g}\text{}{\mathrm{\:day}}^{-1}\mathrm{)=\:}\frac{\mathrm{Wf} - \mathrm{Wi}}{t}$$

Where: Wf is the final weight and Wi is the initial weight and t is the time in days.

Feed Conversion Ratio

4 $$\:\mathrm{FCR=}\mathrm{grams\:of\:feed\:consumed}\:/\mathrm{grams\:of\:biomass\:increase}$$

Protein Efficiency

5 $$\:\mathrm{PE=}\mathrm{grams\:of\:biomass\:increase}/\mathrm{grams\:of\:protein\:ingested\:}$$

6 $$\:\mathrm{Specific} \; \mathrm{Growth} \; \mathrm{Rate}\text{}\mathrm{SGR\:=\:}\frac{(\mathrm{InWf-\:InWi)}}{t}$$

Where: $\:\mathrm{InWf}\mathrm{\:is\:the\:natural\:logarithm\:of\:the\:final\:weight}$, $\:\mathrm{InWi}\text{}$is the natural logarithm of the initial weight and **t** is the time in days

7 $$\:\mathrm{F}\mathrm{i}\mathrm{s}\mathrm{h}\:\mathrm{S}\mathrm{u}\mathrm{r}\mathrm{v}\mathrm{i}\mathrm{v}\mathrm{a}\mathrm{l}\:\mathrm{R}\mathrm{a}\mathrm{t}\mathrm{e}\:\:\:\mathrm{F}\mathrm{SR\:}\left(\mathrm{\%}\right)\mathrm{=}\frac{\mathrm{\:Final\:EquationNumber\:of\:animals}}{\mathrm{Initial\:EquationNumber\:of\:animals\:}} \times {100}$$

8 $$\:\mathrm{Total\:}\mathrm{Fruit} \;\; \mathrm{Yield} \;\; \mathrm{TFY}\mathrm{(kg}\text{}{\mathrm{m}}^{-2}\mathrm{)\:=\:}\frac{kilograms\:of\:fruit}{Area\:used\:{m}^{2}}$$

Dry Weight

9 $$\:\text{}\mathrm{DW\:(g)\:=\:Plant\:weight\:after\:dehydration\:for\:72\:hours\:at\:} 70 ^{\circ} {\mathrm{C}}$$

10 $$\:\mathrm{R}\mathrm{e}\mathrm{l}\mathrm{a}\mathrm{t}\mathrm{i}\mathrm{v}\mathrm{e}\:\mathrm{G}\mathrm{r}\mathrm{o}\mathrm{w}\mathrm{t}\mathrm{h}\:\mathrm{R}\mathrm{a}\mathrm{t}\mathrm{e}\:\mathrm{R}\mathrm{G}\mathrm{R}\:\left(\mathrm{g}\:{\mathrm{\:g}}^{-1}\:{\mathrm{day}}^{-1}\right)\:=\:\frac{(\mathrm{I}\mathrm{n}\mathrm{D}\mathrm{W}\mathrm{f}\:-\:\mathrm{I}\mathrm{n}\mathrm{D}\mathrm{W}\mathrm{i})}{t}$$

Where: InDWf is the natural logarithm of the final dry weight, InDWi is the natural logarithm of the initial dry weight and t is the time in days

11 $$\:\mathrm{Plant} \; \mathrm{Survival} \; \mathrm{Rate}\; \mathrm{PSR\:}\left(\mathrm{\%}\right)\mathrm{=\:}\frac{\mathrm{F}\mathrm{i}\mathrm{n}\mathrm{a}\mathrm{l}\:\mathrm{n}\mathrm{u}\mathrm{m}\mathrm{b}\mathrm{e}\mathrm{r}\:\mathrm{o}\mathrm{f}\:\mathrm{p}\mathrm{l}\mathrm{a}\mathrm{n}\mathrm{t}\mathrm{s}}{\mathrm{I}\mathrm{n}\mathrm{i}\mathrm{t}\mathrm{i}\mathrm{a}\mathrm{l}\:\mathrm{n}\mathrm{u}\mathrm{m}\mathrm{b}\mathrm{e}\mathrm{r}\:\mathrm{o}\mathrm{f}\:\mathrm{p}\mathrm{l}\mathrm{a}\mathrm{n}\mathrm{t}\mathrm{s}\text{}} \times {100}$$

12 $$\:\mathrm{C}\mathrm{r}\mathrm{o}\mathrm{p}\:\mathrm{G}\mathrm{r}\mathrm{o}\mathrm{w}\mathrm{t}\mathrm{h}\:\mathrm{R}\mathrm{a}\mathrm{t}\mathrm{e}\:\:\:\:\:\mathrm{C}\mathrm{G}\mathrm{R}\:(\mathrm{g}\mathrm{c}\mathrm{m}^{-2}{\mathrm{\:day}}^{-1})=\frac{\left(1\right)({\mathrm{D}\mathrm{W}}_{2}-\:{\mathrm{D}\mathrm{W}}_{1})}{\left(\mathrm{S}\mathrm{S}\right)({\mathrm{t}}_{2}-\:{\mathrm{t}}_{1})}$$

Where: DW_2_, DW_1_ is the final and initial dry weight of the plant; SS is the soil surface expressed in cm^2^; t_2_, t_1_ time in days.

####  Environmental variables

Environmental performance was evaluated using indicators related to water consumed, water efficiency, nutrient efficiency, energy demand, and greenhouse gas emissions. These variables were considered: water consumed (defined as the total volume of water associated with each system over the entire production cycle), water use efficiency (Eq. 13), carbon footprint^19^, energy consumption (calculated from electrical consumption), Nitrogen use efficiency (Eq. 14), Phosphorus use efficiency (Eq. 15) and Potassium use efficiency (Eq. 16). For the aquaculture module (AM) and hydroponic module (HM), a modified version of the nutrient efficiency formulas was used to reflect the lack of integration between fish and plants (Eqs. 17, 18, 19, 20, 21 and 22).

13 $$\:\mathrm{Water}\mathrm{\:Use\:}\mathrm{Efficiency} \; \; \mathrm{W}\mathrm{UE}\text{}\left(\mathrm{\%}\right)\mathrm{=\:}\frac{\mathrm{K}\mathrm{i}\mathrm{l}\mathrm{o}\mathrm{g}\mathrm{r}\mathrm{a}\mathrm{m}\mathrm{s}\:\mathrm{o}\mathrm{f}\:\mathrm{f}\mathrm{r}\mathrm{u}\mathrm{i}\mathrm{t}\:\mathrm{o}\mathrm{r}\:\mathrm{t}\mathrm{i}\mathrm{l}\mathrm{a}\mathrm{p}\mathrm{i}\mathrm{a}}{{m}^{3\:}\mathrm{o}\mathrm{f}\:\mathrm{w}\mathrm{a}\mathrm{t}\mathrm{e}\mathrm{r}\:\mathrm{c}\mathrm{o}\mathrm{n}\mathrm{s}\mathrm{u}\mathrm{m}\mathrm{e}\mathrm{d}}$$

14 $$\:\mathrm{N}\mathrm{i}\mathrm{t}\mathrm{r}\mathrm{o}\mathrm{g}\mathrm{e}\mathrm{n}\:\mathrm{U}\mathrm{s}\mathrm{e}\:\mathrm{E}\mathrm{f}\mathrm{f}\mathrm{i}\mathrm{c}\mathrm{i}\mathrm{e}\mathrm{n}\mathrm{c}\mathrm{y}\:\left(NUE\right)=\:\:\frac{{\mathrm{N}}_{\mathrm{p}\mathrm{l}\mathrm{a}\mathrm{n}\mathrm{t}}+\:{\mathrm{N}}_{\mathrm{f}\mathrm{i}\mathrm{s}\mathrm{h}}}{\mathrm{F}\mathrm{N}\:\mathrm{*}\:\mathrm{M}\mathrm{F}\:\mathrm{*}\:\mathrm{T}}$$

Where: FN is nitrogen content in feed (g g⁻¹); MF = feeding rate (g day⁻¹); T is the time in days; N_fish is nitrogen retained in fish (g) and N_plant is nitrogen assimilated by plants (g).

15 $$\:\:\:\mathrm{P}\mathrm{h}\mathrm{o}\mathrm{s}\mathrm{p}\mathrm{h}\mathrm{o}\mathrm{r}\mathrm{u}\mathrm{s}\:\mathrm{U}\mathrm{s}\mathrm{e}\:\mathrm{E}\mathrm{f}\mathrm{f}\mathrm{i}\mathrm{c}\mathrm{i}\mathrm{e}\mathrm{n}\mathrm{c}\mathrm{y}\:\left(PUE\right)=\:\:\frac{{\mathrm{P}}_{\mathrm{p}\mathrm{l}\mathrm{a}\mathrm{n}\mathrm{t}}+\:{\mathrm{P}}_{\mathrm{f}\mathrm{i}\mathrm{s}\mathrm{h}}}{\mathrm{F}\mathrm{P}\:\mathrm{*}\:\mathrm{M}\mathrm{F}\:\mathrm{*}\:\mathrm{T}}$$

Where: FP is phosphorus content in feed (g g⁻¹); MF = feeding rate (g day⁻¹); T is the time in days; P_fish is phosphorus retained in fish(g) and P_plant is phosphorus assimilated by plants(g).

16 $$\:\mathrm{P}\mathrm{o}\mathrm{t}\mathrm{a}\mathrm{s}\mathrm{s}\mathrm{i}\mathrm{u}\mathrm{m}\:\mathrm{U}\mathrm{s}\mathrm{e}\:\mathrm{E}\mathrm{f}\mathrm{f}\mathrm{i}\mathrm{c}\mathrm{i}\mathrm{e}\mathrm{n}\mathrm{c}\mathrm{y}\:\left(KUE\right)=\:\:\frac{{K}_{\mathrm{p}\mathrm{l}\mathrm{a}\mathrm{n}\mathrm{t}}+\:{K}_{\mathrm{f}\mathrm{i}\mathrm{s}\mathrm{h}}}{\mathrm{F}\mathrm{K}\:\mathrm{*}\:\mathrm{M}\mathrm{F}\:\mathrm{*}\:\mathrm{T}}$$

Where: FK is potassium content in feed (g g⁻¹); MF = feeding rate (g day⁻¹); T is the time in days; K_fish is potassium retained in fish(g) and K_plant is potassium assimilated by plants(g).

Where: FN, FP and FK are the Nitrogen, Phosphorus and Potassium content in the fish feed (gN/g, gP/g and gK/g)) respectively, MF is the feeding rate (g/day), T is the duration of production (days), N_plant_, P_plant_ and K_plant_ are the average nitrogen, phosphorus and potassium assimilated by the plants at harvest (g), N_fish_, P_fish_ and K_fish_ are the average nitrogen, phosphorus and potassium incorporated in the fish (g).

17 $$\:\mathrm{N}\mathrm{i}\mathrm{t}\mathrm{r}\mathrm{o}\mathrm{g}\mathrm{e}\mathrm{n}\:\mathrm{U}\mathrm{s}\mathrm{e}\:\mathrm{E}\mathrm{f}\mathrm{f}\mathrm{i}\mathrm{c}\mathrm{i}\mathrm{e}\mathrm{n}\mathrm{c}\mathrm{y}\:\left({NUE}_{AM}\right)=\:\:\frac{{\mathrm{N}}_{\mathrm{f}\mathrm{i}\mathrm{s}\mathrm{h}}}{\mathrm{F}\mathrm{N}\:\mathrm{*}\:\mathrm{M}\mathrm{F}\:\mathrm{*}\:\mathrm{T}}$$

Where: FN is nitrogen content in feed (g g⁻¹); MF = feeding rate (g day⁻¹); T is the time in days and N_fish is nitrogen retained in fish (g).

18 $$\:\mathrm{P}\mathrm{h}\mathrm{o}\mathrm{s}\mathrm{p}\mathrm{h}\mathrm{o}\mathrm{r}\mathrm{u}\mathrm{s}\:\mathrm{U}\mathrm{s}\mathrm{e}\:\mathrm{E}\mathrm{f}\mathrm{f}\mathrm{i}\mathrm{c}\mathrm{i}\mathrm{e}\mathrm{n}\mathrm{c}\mathrm{y}\:\left({PUE}_{AM}\right)=\:\:\frac{{\mathrm{P}}_{\mathrm{f}\mathrm{i}\mathrm{s}\mathrm{h}}}{\mathrm{F}\mathrm{P}\:\mathrm{*}\:\mathrm{M}\mathrm{F}\:\mathrm{*}\:\mathrm{T}}$$

Where: FP is phosphorus content in feed (g g⁻¹); MF = feeding rate (g day⁻¹); T is the time in days and P_fish is phosphorus retained in fish (g).

19 $$\:\mathrm{P}\mathrm{o}\mathrm{t}\mathrm{a}\mathrm{s}\mathrm{s}\mathrm{i}\mathrm{u}\mathrm{m}\:\mathrm{U}\mathrm{s}\mathrm{e}\:\mathrm{E}\mathrm{f}\mathrm{f}\mathrm{i}\mathrm{c}\mathrm{i}\mathrm{e}\mathrm{n}\mathrm{c}\mathrm{y}\:\left({KUE}_{AM}\right)=\:\:\frac{\:{\mathrm{K}}_{\mathrm{f}\mathrm{i}\mathrm{s}\mathrm{h}}}{\mathrm{F}\mathrm{K}\:\mathrm{*}\:\mathrm{M}\mathrm{F}\:\mathrm{*}\:\mathrm{T}\:\:}$$

Where: FK is potassium content in feed (g g⁻¹); MF = feeding rate (g day⁻¹); T is the time in days and K_fish is potassium retained in fish (g).

20 $$\:\mathrm{N}\mathrm{i}\mathrm{t}\mathrm{r}\mathrm{o}\mathrm{g}\mathrm{e}\mathrm{n}\:\mathrm{U}\mathrm{s}\mathrm{e}\:\mathrm{E}\mathrm{f}\mathrm{f}\mathrm{i}\mathrm{c}\mathrm{i}\mathrm{e}\mathrm{n}\mathrm{c}\mathrm{y}\:\left({NUE}_{HM}\right)=\:\:\frac{\mathrm{N}\:\mathrm{a}\mathrm{s}\mathrm{s}\mathrm{i}\mathrm{m}\mathrm{i}\mathrm{l}\mathrm{a}\mathrm{t}\mathrm{e}\mathrm{d}\:\mathrm{i}\mathrm{n}\:\mathrm{t}\mathrm{h}\mathrm{e}\:\mathrm{p}\mathrm{l}\mathrm{a}\mathrm{n}\mathrm{t}\:\left(\mathrm{g}\right)}{\mathrm{N}\:\mathrm{s}\mathrm{u}\mathrm{p}\mathrm{p}\mathrm{l}\mathrm{i}\mathrm{e}\mathrm{d}\:\mathrm{t}\mathrm{o}\:\mathrm{t}\mathrm{h}\mathrm{e}\:\mathrm{s}\mathrm{o}\mathrm{l}\mathrm{u}\mathrm{t}\mathrm{i}\mathrm{o}\mathrm{n}\:\left(\mathrm{g}\right)} \times 100$$

21 $$\:\mathrm{P}\mathrm{h}\mathrm{o}\mathrm{s}\mathrm{p}\mathrm{h}\mathrm{o}\mathrm{r}\mathrm{u}\mathrm{s}\:\mathrm{U}\mathrm{s}\mathrm{e}\:\mathrm{E}\mathrm{f}\mathrm{f}\mathrm{i}\mathrm{c}\mathrm{i}\mathrm{e}\mathrm{n}\mathrm{c}\mathrm{y}\:\left({PUE}_{HM}\right)=\frac{\mathrm{P}\:\mathrm{a}\mathrm{s}\mathrm{s}\mathrm{i}\mathrm{m}\mathrm{i}\mathrm{l}\mathrm{a}\mathrm{t}\mathrm{e}\mathrm{d}\:\mathrm{i}\mathrm{n}\:\mathrm{t}\mathrm{h}\mathrm{e}\:\mathrm{p}\mathrm{l}\mathrm{a}\mathrm{n}\mathrm{t}\:\:\left(\mathrm{k}\mathrm{g}\right)}{\mathrm{P}\:\mathrm{s}\mathrm{u}\mathrm{p}\mathrm{p}\mathrm{l}\mathrm{i}\mathrm{e}\mathrm{d}\:\mathrm{t}\mathrm{o}\:\mathrm{t}\mathrm{h}\mathrm{e}\:\mathrm{s}\mathrm{o}\mathrm{l}\mathrm{u}\mathrm{t}\mathrm{i}\mathrm{o}\mathrm{n}\:\left(\mathrm{g}\right)} \times 100$$

22 $$\:\mathrm{P}\mathrm{o}\mathrm{t}\mathrm{a}\mathrm{s}\mathrm{s}\mathrm{i}\mathrm{u}\mathrm{m}\:\mathrm{U}\mathrm{s}\mathrm{e}\:\mathrm{E}\mathrm{f}\mathrm{f}\mathrm{i}\mathrm{c}\mathrm{i}\mathrm{e}\mathrm{n}\mathrm{c}\mathrm{y}\:\left({KUE}_{HM}\right)=\frac{\mathrm{K}\:\mathrm{a}\mathrm{s}\mathrm{s}\mathrm{i}\mathrm{m}\mathrm{i}\mathrm{l}\mathrm{a}\mathrm{t}\mathrm{e}\mathrm{d}\:\mathrm{i}\mathrm{n}\:\mathrm{t}\mathrm{h}\mathrm{e}\:\mathrm{p}\mathrm{l}\mathrm{a}\mathrm{n}\mathrm{t}\:\:\left(\mathrm{k}\mathrm{g}\right)}{\mathrm{K}\:\mathrm{s}\mathrm{u}\mathrm{p}\mathrm{p}\mathrm{l}\mathrm{i}\mathrm{e}\mathrm{d}\:\mathrm{t}\mathrm{o}\:\mathrm{t}\mathrm{h}\mathrm{e}\:\mathrm{s}\mathrm{o}\mathrm{l}\mathrm{u}\mathrm{t}\mathrm{i}\mathrm{o}\mathrm{n}\:\left(\mathrm{g}\right)} \times 100$$

#### Economic variables

To assess the financial viability and profitability of each production system, three key economic variables were calculated: Total Operating Cost (TOC), Gross Income (GI), and Net Profit (NP). Total operating cost included expenses associated with feed input, energy consumption, and routine system operation. Gross income was estimated based on the market value of harvested fish and plant biomass at the end of the production cycle. Net profit was Net profit was calculated according to Eq. (23):

23 $${\text{Net Profit}} \; {\mathrm{NP}}= \mathrm{GI}- \mathrm{TOC}$$

###  Data analysis

Statistical analyses were performed using JMP^®^ software (version 9.0.1). The experimental unit was defined as the production system (IAS, AM, and HM). Data obtained from the three production cycles conducted in different years were treated as temporal repetitions of the same experimental design rather than as independent biological replicates. Prior to analysis, data were tested for normality and homogeneity of variance using the Shapiro–Wilk and Levene tests, respectively. When assumptions were met, differences among systems were evaluated using one-way analysis of variance (ANOVA), followed by Tukey’s post hoc test. Results are reported as mean ± standard deviation, and statistical significance was assessed at a confidence level of *P* < 0.05.

### Bioeconomic Performance Index (BPI): formulation, normalization, and assumptions

The Bioeconomic Performance Index (BPI) was developed as a comparative, system-level indicator to integrate productive, environmental, and economic performance across the evaluated production systems. The analysis was restricted to on-site operational processes occurring within a single 180-day production cycle and considered only direct inputs and outputs associated with fish and plant production. Upstream processes such as feed and fertilizer manufacturing, infrastructure construction, and downstream processes including transport and commercialization were excluded from the system boundaries.

The BPI integrates benefit-related indicators: total yield (R), nitrogen use efficiency (NUE), phosphorus use efficiency (PUE), potassium use efficiency (KUE), and net profit (NP) and resource-related indicators: water consumption (WC), energy consumption (EN), carbon footprint (CF), and area used (AU). All indicators were assigned equal weights to ensure a transparent, parsimonious, and reproducible multi-criteria comparison, avoiding subjective prioritization among heterogeneous variables. Prior to aggregation, all indicators were normalized using min–max normalization to transform values into dimensionless scores ranging from 0 to 1. For indicators where higher values indicate improved performance (benefit-related indicators), normalization was performed as:

24 $$\:\mathrm{Normalization\:of\:benefit-related\:indicators\:\:(X`)}\mathrm{=}\frac{\mathrm{X}\:-\:{\mathrm{X}}_{\mathrm{m}\mathrm{i}\mathrm{n}}}{{\mathrm{X}}_{\mathrm{m}\mathrm{a}\mathrm{x}}-\:{\mathrm{X}}_{\mathrm{m}\mathrm{i}\mathrm{n}}}$$

Where: X is the observed value of the indicator, and X_min_ and X_max_ are the minimum and maximum values of that indicator across the evaluated systems.

For indicators where lower values represent improved performance (resource-related indicators), normalization was performed as:

25 $$\:\mathrm{Normalization\:of\:resource-related\:indicators\:\:(X`}\mathrm{)=}\frac{{\mathrm{X}}_{\mathrm{m}\mathrm{a}\mathrm{x}\:}-\:\mathrm{X}\:\:}{{\mathrm{X}}_{\mathrm{m}\mathrm{a}\mathrm{x}}-\:{\mathrm{X}}_{\mathrm{m}\mathrm{i}\mathrm{n}}}$$

Where: X is the observed value of the indicator, and X_min_ and X_max_ are the minimum and maximum values of that indicator across the evaluated systems.

After normalization, the BPI was calculated as a ratio between the mean of normalized benefit-related indicators and the mean of normalized resource-related indicators, preserving the conceptual structure of a benefit-to-resource index while avoiding implicit weighting caused by unequal numbers of variables:

26 $$\:\mathrm{B}\mathrm{i}\mathrm{o}\mathrm{e}\mathrm{c}\mathrm{o}\mathrm{n}\mathrm{o}\mathrm{m}\mathrm{i}\mathrm{c}\:\mathrm{P}\mathrm{e}\mathrm{r}\mathrm{f}\mathrm{o}\mathrm{r}\mathrm{m}\mathrm{a}\mathrm{n}\mathrm{c}\mathrm{e}\:\mathrm{I}\mathrm{n}\mathrm{d}\mathrm{e}\mathrm{x}\:\left(\mathrm{B}\mathrm{P}\mathrm{I}\right)\:=\frac{\frac{1}{5}\:(\mathrm{R`}+\mathrm{N}\mathrm{U}\mathrm{E`}+\mathrm{P}\mathrm{U}\mathrm{E`}+\mathrm{K}\mathrm{U}\mathrm{E`}+\mathrm{N}\mathrm{P`})}{\frac{1}{4}\:(\mathrm{W}\mathrm{C`}\:+\:\mathrm{E}\mathrm{N`}\:+\:\mathrm{C}\mathrm{F`}\:+\:\mathrm{A}\mathrm{U})\:}$$

Where: R` is total yield; NUE` is efficiency in the use of nitrogen; PUE` is efficiency in the use of phosphorus; KUE` is efficiency in the use of potassium; NP` is net profit correspond to normalized benefit-related indicators. WC` is water consumption; EN` is energy consumption; CF` is carbon footprint and AU` is area used correspond to normalized resource-related indicators. All variables are dimensionless and range between 0 and 1.

Because of its ratio-based formulation, BPI values are not constrained to a maximum of 1. Values greater than 1 indicate that normalized benefits outweigh normalized resources, whereas values lower than 1 indicate the opposite. The BPI is intended as a relative decision-support indicator applicable only within the defined experimental boundaries and does not aim to replace full life cycle assessment or broader sustainability frameworks.

The primary measured variables used as inputs for all equations and normalization procedures are listed in Appendix A to facilitate transparency and reproducibility.

### Sensitivity analysis

A one-factor-at-a-time sensitivity analysis was conducted to evaluate the robustness of the Bioeconomic Performance Index and to identify parameters exerting the greatest influence on system performance. The analysis was performed using the intensive aquaponic system (IAS) as the reference case. Key variables were selected based on their relevance in aquaponic production systems and because they represent major sources of operational and economic uncertainty, including energy consumption, feed input, and output market prices. Each parameter was independently varied by ± 20% relative to its observed baseline value, while all other variables were held constant. The resulting changes in the Bioeconomic Performance Index (BPI) were quantified to assess system sensitivity and to verify that the relative ranking of production systems was not driven by a single variable.

## Results and discussion

### Water quality

Water quality variables showed significant differences between the systems evaluated (Table 4). Temperature remained relatively stable between treatments (25.8–26.5 °C), with no significant differences (*P* > 0.05), remaining within the optimal range for the development of *Oreochromis niloticus* and *Solanum lycopersicum*^20^. Dissolved oxygen (DO) levels were slightly higher in the aquaponic system (IAS) and the aquaculture module (AM) compared to the hydroponic module (HM), although the differences were not statistically significant. pH values ​​varied between systems. In the HM system, the nutrient solution was prepared with a pH of 6.0, as recommended in the fertilizer’s technical data sheet (Peters^®^ 5–11-26). This acidity value was kept constant throughout the experiment (6.1 ± 0.2), ensuring nutrient solubility. In contrast, the IAS and AM systems required continuous monitoring and frequent pH adjustments to maintain stability. Despite the fluctuations, the recorded pH values ​​remained within the optimal physiological range, ensuring adequate conditions for both tilapia metabolism and nutrient availability for plants and nitrifying bacteria^21^.

Nitrogen compounds also showed significant differences among systems. In the IAS, lower nitrate concentrations compared to the AM reflect the combined effects of efficient nitrification supported by continuous recirculation and biofiltration, together with continuous plant uptake acting as an additional nutrient sink. In contrast, nitrate accumulation in the AM is expected when feed-derived nitrogen is nitrified without a plant uptake pathway. Non-ionized ammonia followed a similar pattern, with lower concentrations in the IAS (0.51 ± 0.13 mg/L) than in the AM (0.96 ± 0.14 mg/L), indicating reduced exposure of fish to nitrogenous waste and more stable nitrogen transformation within the integrated system^22^. Compared to the AM, lower ammonia and nitrate concentrations in the IAS reduce the nitrogen load processed by the biofilter and limit fish exposure to potentially stressful nitrogenous compounds. From a functional perspective, this condition favors a more stable microbial nitrification process and reduces physiological stress in fish, which may contribute to improved growth performance observed in the integrated system. In contrast, the AM relies solely on biofiltration for nitrogen removal, increasing the likelihood of nitrogen accumulation under intensive feeding conditions^23^.

Phosphate and potassium levels were highest in the HM (43.42 ± 5.87 mg/L and 181.93 ± 19.21 mg/L, respectively), consistent with the composition of the nutrient solution. The IAS presented intermediate values (P: 5.21 ± 0.63 mg/L; K: 80.59 ± 9.73 mg/L), attributable to the mineralization of organic matter and nutrient cycling, while the AM presented slightly elevated values due to food inputs (P: 7.5 ± 0.11 mg/L; K: 93.82 ± 7.48 mg/L), but with lower assimilation due to the absence of plants^24^. Electrical conductivity (EC) was notably higher in the HM (3.4 ± 0.3 mS/cm), due to the ion concentration in the nutrient solution. The IAS maintained moderate EC values (2.1 ± 0.3 mS/cm), reflecting partial nutrient reutilization and biological uptake, while the AM showed the lowest EC (1.8 ± 0.2 mS/cm), due to the exclusive presence of fish and the absence of mineral recovery by plants. These results confirm that aquaponic systems promote improved water quality stability and nutrient recycling, reducing the accumulation of waste compounds and supporting a more integrated and sustainable production model. This is consistent with the findings of Goddek et al^1^., Zou et al^2^., Forchino et al^5^. and Subramanian et al^25^., who highlight the synergistic advantages of closed-loop aquaponic systems. Moreover, the reduction of nitrate in the IAS likely benefited fish health; elevated nitrate levels in recirculating systems can impair tilapia growth and health^26^, so the plant-driven nutrient removal in aquaponics is advantageous.

In the integrated aquaponic system (IAS), the coupling of fish and plants created additional biological nutrient sinks and enhanced nitrification, resulting in lower nitrate and non-ionized ammonia concentrations compared to the fish-only aquaculture module (AM). In contrast, the AM lacked plant-mediated nutrient assimilation, leading to greater accumulation of nitrogenous compounds^22^. Phosphate and potassium concentrations were highest in the hydroponic module (HM) due to the use of fertilizer-based nutrient solutions, intermediate in the AM where nutrients derived from feed accumulated without plant uptake, and lowest in the IAS due to continuous nutrient assimilation and recycling by plants^27^. Electrical conductivity reflected these patterns, with higher values in HM due to elevated ionic inputs, lower values in AM due to limited external nutrient inputs, and intermediate values in IAS representing a balance between nutrient generation from fish metabolism and removal by plants. Overall, system integration improved water quality stability and nutrient recycling while reducing the accumulation of potentially harmful compounds^1,4,5^.

Overall, differences in water quality among systems reflect the combined influence of system design, microbial nitrification dynamics, and nutrient uptake efficiency rather than a single controlling factor^28^. In the IAS, the integration of biological components promotes continuous nutrient transformation and removal, resulting in more stable water quality conditions that directly influence fish physiological performance and plant nutrient availability discussed in subsequent sections^29^. Differences among systems were evaluated using one-way ANOVA followed by Tukey’s post hoc test and were considered statistically significant at *P* < 0.05.

**Table 4:** Water quality values measured in fish tanks from the Intensive Aquaponic System (IAS), the Aquaculture Module (AM) and the Hydroponic Module (HM).

Variable	IAS	AM	HM
Temperature (ºC)	26.1 ± 1.2^a^	26.5 ± 1.5^a^	25.8 ± 1.3^a^
Dissolved oxygen (mg/L)	5.84 ± 0.43^a^	5.24 ± 0.68^a^	4.05 ± 0.36^b^
pH	6.9 ± 0.3^a^	7.1 ± 0.4^a^	6.1 ± 0.2^b^
Nitrates (mg/L)	65.05 ± 8.34^c^	101.02 ± 10.87^b^	134.02 ± 13.03^a^
Nitrites (mg/L)	1.13 ± 0.76^b^	2.92 ± 0.85^a^	–
Non-ionized ammonia (mg/L)	0.51 ± 0.13^b^	0.96 ± 0.14 ^a^	–
Phosphate (mg/L)	5.21 ± 0.63^c^	7.5 ± 0.11 ^b^	43.42 ± 5.87^a^
Potassium (mg/L)	80.59 ± 9.73^bc^	93.82 ± 7.48 ^b^	181.93 ± 19.21^a^
Electrical conductivity (mS)	2.1 ± 0.3^b^	1.8 ± 0.2 ^bc^	3.4 ± 0.3^a^

Values are presented as mean ± standard deviation of samples collected during the experimental period. Values with different superscripts present significant differences (*P* < 0.05).

### Productive performance

The productive performance of Nile tilapia (*Oreochromis niloticus*) differed among systems (Table 5). The Intensive Aquaponic System (IAS) achieved the highest total tilapia yield (TTY), this performance is consistent with the more stable water quality conditions associated with plant integration in aquaponic systems^30^. Previous studies have reported similar trends. Goddek et al^1^. demonstrated that integrated aquaponic systems reduced nitrogenous waste accumulation and improved fish growth stability compared to standalone aquaculture. Similarly, Yep and Zheng^3^ reported lower feed conversion ratios and improved growth performance in tilapia cultured under integrated nutrient recycling systems, attributing these effects to improved water quality and reduced physiological stress.

Total weight gain (TWG) and daily weight gain (DWG) were also higher in the IAS, indicating more favorable growth dynamics under integrated conditions. Similarly, the specific growth rate (SGR) was slightly higher in the IAS than in the aquaculture module (AM), suggesting improved feed assimilation in the integrated system^31^. Feed conversion ratio (FCR) was lower in the IAS (1.38) compared to the AM (1.55), and protein efficiency (PE) was correspondingly higher, reflecting more efficient utilization of dietary inputs. This improvement in feed conversion efficiency can be mechanistically linked to reduced physiological stress under integrated conditions, where lower nitrogenous waste concentrations and more stable dissolved oxygen levels decrease maintenance energy requirements^32,33^. As a result, a greater proportion of dietary energy and protein is allocated to growth rather than to stress responses, leading to improved FCR and PE.

Survival rate (FSR) was also marginally higher in the IAS, consistent with reduced environmental stress and improved system stability under integrated operation^34^. From a physiological perspective, improved tilapia growth in the IAS can be linked to lower exposure to non-ionized ammonia and nitrate concentrations approaching sublethal stress thresholds^35^. Chronic exposure to elevated ammonia is known to impair gill function, reduce oxygen uptake efficiency, and increase maintenance energy expenditure, while prolonged exposure to high nitrate concentrations can negatively affect growth performance and feed utilization^26^. In the IAS, reduced nitrogenous waste concentrations together with more stable dissolved oxygen conditions likely decreased metabolic stress, allowing a greater proportion of assimilated energy to be allocated to somatic growth rather than maintenance metabolism^36^. Overall, these results indicate that aquaponic integration can support efficient tilapia growth and feed utilization under intensive stocking conditions, achieving performance comparable to or slightly superior to conventional aquaculture systems. However, these benefits are closely linked to the maintenance of stable water quality and effective system management, highlighting that biological advantages in aquaponics are contingent on adequate operational control rather than being inherent to system integration alone.

**Table 5:** Productive performance indicators for fish cultivation in the Intensive Aquaponic System (IAS), and Aquaculture Module (AM).

Variable	IAS	AM	HM
Total tilapia yield (kg/m^3^)	15.3 ± 1.2^a^	14.2 ± 1.5^a^	–
Total weight gain (g)	635.18 ± 10.43^a^	561.6 ± 12.68^b^	–
Daily weight gain (g/day)	3.53 ± 0.23^a^	3.12 ± 0.14^b^	–
Feed conversion ratio	1.6 ± 0.13^b^	2.1 ± 0.07^a^	–
Protein efficiency	2.41 ± 0.15^a^	2.17 ± 0.08^b^	–
Specific growth rate (%)	1.95 ± 0.13^ab^	1.86 ± 0.14^a^	–
Condition factor	1.57 ± 0.16^a^	1.33 ± 0.18^ab^	–
Fish survival rate (%)	92.5 ± 0.13^a^	89.6 ± 0.14^b^	–

Values are presented as mean ± standard deviation of samples collected during the experimental period. Values with different superscripts present significant differences (*P* < 0.05).

Tomato performance differed among the evaluated systems (Table 6). The hydroponic module (HM) exhibited the highest total fruit yield and aboveground biomass, whereas the intensive aquaponic system (IAS) showed lower but still substantial tomato production. These differences were statistically significant (*P* < 0.05) and reflect contrasting nutrient supply strategies between systems.

In the HM, tomato plants were continuously supplied with a complete and balanced nutrient solution, which resulted in higher fruit yield and biomass accumulation. In contrast, tomato production in the IAS relied exclusively on nutrients recovered from aquaculture effluent, without external fertilizer inputs^37^. Despite this limitation, the IAS sustained consistent fruit production, indicating effective nutrient recovery and plant uptake under integrated conditions^1,2^. Differences in tomato yield between the IAS and HM are therefore more closely related to nutrient availability and composition than to plant density, irrigation regime, or greenhouse environmental conditions, which were comparable across systems. In aquaponic systems, nutrient supply is intrinsically linked to fish feed composition and microbial transformation processes, which may constrain the availability of certain nutrients when compared to hydroponic formulations optimized for crop demand^3,5^.

In particular, limitations in root-zone availability of micronutrients such as iron (Fe), magnesium (Mg), and calcium (Ca) have been widely reported in aquaponic cultivation and may partially explain the lower tomato yield observed in the IAS relative to the HM. These micronutrients are essential for photosynthesis, fruit development, and cell wall stability, and their reduced availability can affect yield and fruit uniformity even when macronutrient supply is adequate^8^. Overall, the plant performance results indicate that while hydroponic cultivation maximized tomato yield through targeted nutrient supplementation, the aquaponic system achieved productive tomato cultivation through internal nutrient recycling alone. This highlights a fundamental trade-off between yield maximization and resource circularity, positioning the IAS as a system optimized for nutrient recovery and integrated production rather than maximal horticultural output^7^.

**Table 6:** Productive performance indicators for tomato cultivation in the Intensive Aquaponic System (IAS), and Hydroponic Module (HM).

Variable	IAS	AM	HM
Total fruit yield (kg/m^2^)	6.25 ± 0.23^b^	–	6.89 ± 0.37^a^
Dry weight (g)	390.63 ± 10.83^b^	–	421.55 ± 13.89^a^
Relative growth rate (g/g day)	0.043 ± 0.002^ab^	–	0.045 ± 0.003^a^
Plant survival rate (%)	90.03 ± 0.61^b^	–	96.75 ± 0.83^a^
Crop growth rate (g/cm^2^ day)	0.021 ± 0.002^ab^	–	0.022 ± 0.001^b^

Values are presented as mean ± standard deviation of samples collected during the experimental period. Values with different superscripts present significant differences (*P* < 0.05).

### Environmental performance

Environmental performance indicators differed markedly among the systems (Table 7). The aquaponic system (IAS) exhibited the highest nutrient use efficiency for nitrogen, phosphorus and potassium, significantly surpassing the aquaculture (AM) and hydroponic (HM) modules (*P*< 0.05). This highlights the system’s ability to optimize nutrient recycling, minimizing external losses and improving the capture of available resources. These results are consistent with previous findings by Joyce et al.^38^, Sebastião et al.^39^, Behr et al.^40^ and Félix-Cuencas et al.^41^ which characterize aquaponics as a low-emission, nutrient-efficient model. Water use efficiency (WUE) was also highest in IAS, compared to AM and HM, underscoring the system’s capacity to maximize biomass production per unit of water consumed. This characteristic makes aquaponics especially advantageous in water-limited or arid regions, where integrated systems contribute meaningfully to water conservation^5^. In terms of energy use and environmental emissions, IAS and AM reported similar electricity consumption values, while the HM system consumed significantly less energy due to the absence of fish-related pumping and filtration. Despite higher energy demand, the IAS achieved the highest nutrient recovery and combined productivity, which could justify its energy use. In other words, the resources consumed by the IAS produce proportionally more production (fish + fruit) and less waste, an important factor for sustainability. These trade-offs underscore the need for future improvements, such as the integration of renewable energy or more efficient equipment, to further improve the environmental profile of intensive aquaponic systems^42^. Overall, the environmental analysis confirms the main advantage of aquaponics: it recycles nutrients and water much more efficiently than single-component systems, in line with the principles of the circular economy and sustainable intensification^7^. By producing two types of food with minimal water and nutrient waste, the IAS demonstrated a smaller environmental footprint relative to production, an increasingly crucial metric in modern agriculture.

**Table 7:** Environmental performance indicators of the Intensive Aquaponic System (IAS), Aquaculture Module (AM), and Hydroponic Module (HM).

Variable	IAS	AM	HM
Water consumed (m^3^)	12.4 ± 0.3^b^	9.2 ± 0.5^c^	19.6 ± 0.5^a^
Water use efficiency (%)	58.08 ± 0.88^a^	15.77 ± 0.92^c^	27.36 ± 0.48^b^
Efficiency use of nitrogen (%)	69.05 ± 3.63^a^	42.73 ± 3.11^bc^	50.23 ± 5.87^b^
Efficiency use of phosphorus (%)	66.89 ± 4.73^a^	38.28 ± 5.48^bc^	43.72 ± 5.21^b^
Efficiency use of potassium (%)	78.52 ± 3.48^a^	46.88 ± 2.98^c^	54.34 ± 3.83^b^
Energy consumption (kWh)	607 ± 6^a^	590 ± 4^b^	61 ± 3^c^
Carbon footprint (kg CO_2_-eq)	276.73 ± 17.46^a^	268.98 ± 14.76^a^	37.28 ± 13.62^b^

Values are presented as mean ± standard deviation of samples collected during the experimental period. Values with different superscripts present significant differences (*P* < 0.05).

### Economic performance

Economic performance differed significantly among the evaluated systems (Table 8). The intensive aquaponic system (IAS) recorded the highest gross income as a result of the combined production of fish and tomatoes. However, it also exhibited the highest total operating cost, mainly associated with energy consumption required for continuous water circulation, filtration, and system management, a trend commonly reported for intensive aquaponic configurations^2,8^. Despite these higher costs, the IAS achieved the highest net profit among the evaluated systems, indicating that the additional revenue generated by integrated production compensated for increased operational expenditures.

The higher energy consumption observed in the intensive aquaponic system was primarily associated with the continuous operation of multiple hydraulic and life-support components required to sustain system integration. While this elevated energy use represents an environmental and economic drawback, it is intrinsically linked to the operational requirements necessary to maintain stable water quality, effective nutrient transformation, and biological coupling between fish and plants.

In contrast, the hydroponic module (HM) presented the lowest total operating cost, reflecting lower energy demand and reduced operational complexity. Although its gross income and net profit were substantially lower than those of the IAS, the HM maintained stable economic performance with minimal resource inputs. Similar findings have been reported in standalone hydroponic systems, where simplified management and reduced infrastructure requirements contribute to lower operational costs but also limit overall output^3,43^. The aquaculture module (AM) exhibited moderate operating costs; however, its gross income barely exceeded expenses, resulting in a negligible net profit compared with the other systems, as frequently observed in small-scale or single-species recirculating aquaculture systems^44,45^.

By integrating economic performance with productivity and resource related indicators, the Bioeconomic Performance Index (BPI) provided a composite measure of overall system performance. According to the normalized benefit-to-resource formulation (Eq. 26), the IAS achieved the highest BPI value, indicating that its combined productive and economic benefits outweighed its associated resource use and environmental impacts. The HM showed an intermediate BPI value, reflecting efficient resource use but limited overall output due to the absence of fish production. The AM exhibited the lowest BPI, constrained by lower productivity and higher resources per unit of benefit. These results are consistent with previous comparative assessments using composite sustainability indicators^7,9^.

These results highlight an important trade-off in integrated production systems. While aquaponics involves higher operational complexity and energy demand, the simultaneous production of fish and crops enhances overall resource productivity and economic returns when evaluated using normalized indicators. Similar trade-offs between increased energy use and improved system efficiency have been reported in intensive aquaponic systems operating under controlled conditions^1,2^.

The economic and environmental advantages observed in the intensive aquaponic system arise from the internal coupling of biological and operational processes that enable resource reutilization. Nutrients originating from fish feed are not only converted into fish biomass but are subsequently assimilated by plants, reducing the need for external fertilizer inputs while generating an additional marketable product. This internal nutrient cycling directly explains the higher nutrient use efficiencies observed in the integrated system, as previously described for coupled aquaponic configurations^5,8^.

From an economic perspective, shared infrastructure, water, and energy inputs support two production streams, increasing overall output per unit of resource use. Although the aquaponic system exhibited higher absolute energy consumption, the combined production of fish and crops diluted resource and environmental impacts when assessed through normalized indicators, resulting in a superior bioeconomic performance. This outcome aligns with bioeconomic and sustainability-oriented evaluation frameworks proposed for aquaculture and integrated agri-food systems^7,9^.

From an environmental standpoint, reduced nutrient discharge, improved water use efficiency, and enhanced nutrient recovery collectively lowered the effective impact intensity of the system. These outcomes reflect the synergistic nature of aquaponic systems, where waste streams are transformed into productive inputs, aligning the system with circular economy principles and explaining the concurrent economic gains and environmental impact reduction^1,2,5^.

It is important to note that the economic feasibility of aquaponic systems is strongly influenced by regional conditions, particularly electricity tariffs and local market prices for fish and horticultural products. In regions with high energy costs, the economic advantage of intensive aquaponics may be reduced unless energy efficiency measures or renewable energy sources are implemented. Conversely, in contexts where water scarcity, fertilizer costs, or premium markets for sustainably produced food prevail, the integrated nature of aquaponics may offer a competitive advantage despite higher operational complexity. Therefore, the bioeconomic outcomes reported here should be interpreted within the specific regional and operational context of the study.

**Table 8:** Economic performance indicators of the Intensive Aquaponic System (IAS), Aquaculture Module (AM), and Hydroponic Module (HM).

Variable	IAS	AM	HM
Total operating cost	2261.65 ± 28.63^a^	1841.7 ± 20.11^b^	1017.95 ± 25.87^c^
Gross income	3077.19 ± 19.73^a^	1846.82 ± 17.48^b^	1040.93 ± 19.21^c^
Net profit	815.35 ± 12.73^a^	5.82 ± 2.48^c^	22.05 ± 3.21^b^
Bioeconomic performance index	5.78 ± 0.93^a^	0.19 ± 0.04^bc^	0.24 ± 0.02^b^

Values are presented as mean ± standard deviation of samples collected during the experimental period. Values with different superscripts present significant differences (*P* < 0.05). All monetary values are expressed in Mexican pesos (MXN). The average exchange rate during the study period (Mar 2022 – Aug 2024) was 19.85 MXN/USD.

Sensitivity analysis showed that variations of ± 20% in key parameters did not alter the relative ranking of the evaluated systems according to the Bioeconomic Performance Index. Energy consumption exhibited the highest influence on BPI values, particularly for the intensive aquaponic system, whereas changes in feed input and output market prices produced smaller effects on overall performance. These results indicate that the BPI ranking is robust to plausible operational and economic variability and is not driven by a single parameter.

This study has several limitations that should be acknowledged. Plant nutrient availability in aquaponics was constrained by reliance on fish-derived nutrients, which partially explains the lower tomato yields compared with hydroponics. Energy consumption represented a major operational constraint in the intensive aquaponic system, as confirmed by the sensitivity analysis. In addition, environmental variables such as photoperiod and light intensity were not experimentally manipulated and may influence crop performance. These limitations should be considered when extrapolating the results beyond the evaluated system configuration and scale.

## Conclusion

This study demonstrates that under the evaluated conditions, an intensive aquaponic system integrating tilapia and tomato achieved a higher overall bioeconomic performance than standalone aquaculture and hydroponic systems when productivity, resource use, environmental impact, and economic outcomes were jointly assessed. The application of a normalized Bioeconomic Performance Index revealed that the integrated system effectively balanced higher operational demands with gains in combined output and resource-use efficiency.

Aquaponics improved water and nutrient use efficiency relative to single-component systems, supporting the role of internal nutrient recycling in reducing resource losses. However, this advantage was accompanied by increased energy consumption, which represented the main limiting factor affecting system performance, as confirmed by the sensitivity analysis. While net profit was higher in the aquaponic system, economic benefits remained strongly dependent on energy costs and operational efficiency.

These results indicate that intensive aquaponics can outperform conventional systems in terms of overall bioeconomic performance, but not without trade-offs. The findings are specific to the evaluated system configuration and operational scale and should not be generalized beyond these boundaries. Improvements in energy efficiency, system design, and management practices are essential for enhancing the economic and environmental viability of intensive aquaponic systems.

## Data Availability

The datasets generated during and/or analyzed during the current study are not publicly available due to institutional data protection policies but are available from the corresponding author on reasonable request.

## Appendix: Primary input variables and correspondence with equations

To ensure transparency and reproducibility, Table 9 lists the primary measured variables used in the calculation of all indicators. Each variable is linked to the equations where it is applied.

**Table 9:** Primary input variables, units, and correspondence with equations used in the bioeconomic analysis.

Primary variable	Unit	Description	Reported in	Used in equations
Initial fish biomass	kg	Biomass at stocking	Methods	Growth, FCR, PE
Final fish biomass	kg	Biomass at harvest	Table 5	Growth, FCR, PE, R
Feed supplied	kg	Total feed input	Methods	FCR, PE, NUE, PUE
Tomato yield	kg	Total fruit production	Table 6	R, WUE
Water consumed	m^3^	Total water use	Table 7	WUE, BPI
Energy consumption	kWh	Electricity demand	Table 7	EN, CF, BPI
Carbon emission factor	kg CO₂/kWh	Conversion parameter	Methods	CF
Nitrogen input	kg	Nutrient supplied	Methods	NUE
Nitrogen recovered	kg	Nutrient in biomass	Table 7	NUE
Phosphorus input	kg	Nutrient supplied	Methods	PUE
Phosphorus recovered	kg	Nutrient in biomass	Table 7	PUE
Potassium input	kg	Nutrient supplied	Methods	KUE
Potassium recovered	kg	Nutrient in biomass	Table 7	KUE
Area used	m^2^	System footprint	Methods	AU, BPI
Gross income	MXN	Market value	Table 8	NP, BPI
Operating cost	MXN	Total expenses	Table 8	NP
Indicator maxima/minima	–	Normalization limits	Derived	Equations 24–25

These variables form the complete dataset required to reproduce all 26 equations presented in the Methods section.
